# A Spherical Cap Model of Epidural Hematomas

**DOI:** 10.7759/cureus.53653

**Published:** 2024-02-05

**Authors:** Heng-Chun Liao, Cheng-Loong Liang, Chien-Hua Chen, Chun-Chih Liao, Furen Xiao

**Affiliations:** 1 Department of Medical Imaging, National Taiwan University Hospital, Taipei, TWN; 2 Department of Neurosurgery, E-DA Hospital, Kaohsiung, TWN; 3 Biomedical Engineering, National Taiwan University, Taipei, TWN; 4 Department of Neurosurgery, Taipei Hospital, Taipei, TWN; 5 Medical Device and Imaging, National Taiwan University, Taipei, TWN; 6 Department of Neurosurgery, National Taiwan University Hospital, Taipei, TWN

**Keywords:** computed tomography (ct ), volumetry, abc method, spherical cap, epidural hematoma

## Abstract

Background

Epidural hematomas (EDHs), which have a characteristic biconvex shape, are a type of post-traumatic intracranial mass. EDHs and other types of intracranial hematomas are often diagnosed with computed tomography (CT). The volumes of EDHs are important in treatment decisions and prognosis. Their volumes are usually estimated on CT using the “ABC” method, which is based on the ellipsoid shape rather than their biconvex shape.

Objective

To simulate the biconvex shape, we modeled the geometry of EDHs with two spherical caps. We aim to provide simpler estimation of EDH volumes in clinical settings, and eventually recommend a threshold for surgical evacuation.

Methods

Applying the relationship between the sphere radius, spherical cap height, and base circle radius, we derived formulas for the shape of an EDH, relating its largest diameter and location to the other two diameters. We also estimated EDH volumes using the spherical cap volume and conventional ABC formulas and then constructed a lookup table accordingly.

Results

Validation of the model was performed using 14 CT image sets from previously reported patients with EDHs. Our geometric model demonstrated accurate predictions. The model also allows reducing the number of parameters to be measured in the ABC method from three to one, the hematoma length, showcasing its potential as a reliable tool for clinical decision-making. Based on our model, an EDH longer than 7 cm would occupy more than 30 mL of the intracranial volume.

Conclusion

The proposed model offers a streamlined approach to estimating EDH volumes, reducing the complexity of parameters required for clinical assessments. We recommend a length of 7 cm as a threshold for surgical evacuation of EDHs. This acceleration in decision-making is crucial for managing critically injured patients with traumatic brain injuries. Further validation across diverse patient populations will enhance the generalizability and utility of this geometric modeling approach in clinical settings.

## Introduction

Epidural hematomas (EDHs) are a type of post-traumatic intracranial mass that is often diagnosed with computed tomography (CT) [1]. Other types of hematomas, such as subdural hematomas (SDHs) and intracerebral hematomas (ICHs) are also diagnosed using CT. Although separated by the dura, both EDH and SDH are outside the brain parenchyma and inside the rigid skull. Left untreated, large EDHs and SDHs compress the brain, increase intracranial pressure (ICP), and become life-threatening [2].

On axial CT images, EDHs have a characteristic biconvex shape, differentiating them from crescent-shaped SDHs [3]. The volumes of EDHs are important in treatment decisions and prognosis. Their volumes are usually estimated on CT using the “ABC” method, which is widely applied in measuring the volume of post-traumatic masses, including EDHs, SDHs, and ICHs [4]. However, the "ABC" method is based on the ellipsoid shape rather than the biconvex shape of EDHs.

Previously, we constructed a half-sphere model of the supratentorial brain using CT craniometric data from patients with brain concussion [5]. Using a finite-element method, we simulated brain deformations caused by SDHs and then estimated the ICP [6,7].

In this study, we modeled the geometry of EDHs using two spherical caps (SCs) based on the same model. Because the lesions were located outside the dura, details about the intradural structures could be neglected. The parameters of a SC at any location of the epidural space are related to those of the “ABC” method. Current results were verified with measurements on images reported in our previous publications [8,9].

By simulating the biconvex shape using the spherical model, we aim to provide simpler estimation of EDH volumes in clinical settings, and eventually recommend a threshold for surgical evacuation.

This article was previously posted to the Research Square preprint server on July 15, 2022.

## Materials and methods

Modeling epidural hematoma geometry using two SCs

**Figure 1:**
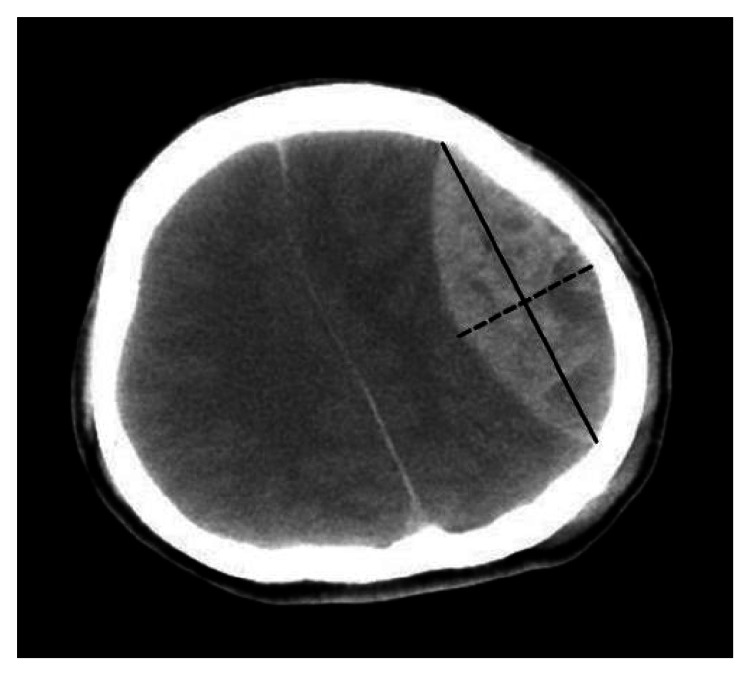
An epidural hematoma on an axial CT image showing its biconvex shape and bilateral symmetry along the long axis (solid line), as well as approximate bilateral symmetry along the short axis (dashed line).

**Figure 2:**
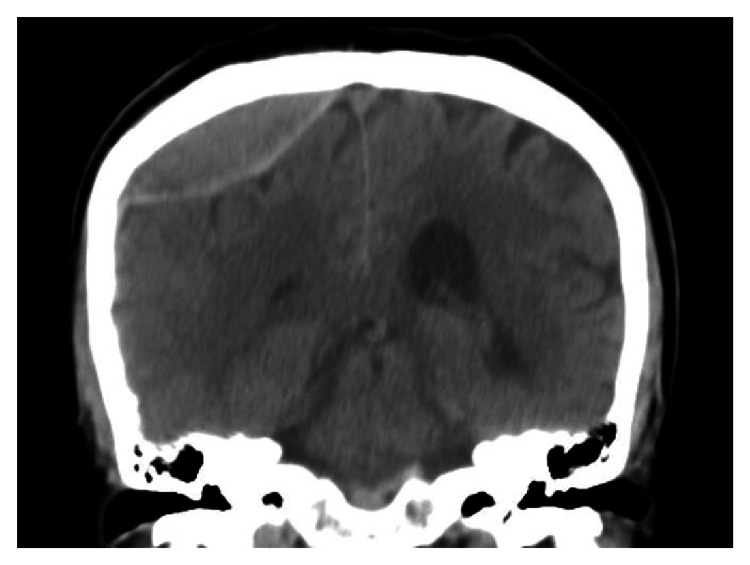
An epidural hematoma on a coronal CT image from a different patient. The image shows its biconvex shape and approximate bilateral symmetry.

On axial and coronal CT images, an EDH typically has a lens-shaped or biconvex configuration (Figures 1, 2). This shape arises because the bleeding occurs between the skull and the dura mater, and as the blood accumulates, it tends to form a convex shape that follows the contours of the skull. Although the actual shape can vary based on the specific circumstances of the injury and the anatomy of the individual, the classic appearance is very common clinically. We therefore developed our model based on the biconvex shape.

**Figure 3:**
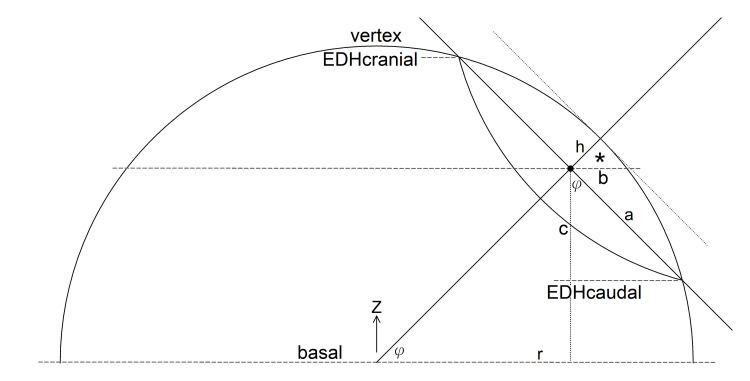
A two-dimensional working plane defined by the Z-axis and the axis of the spherical cap, showing the radius of the base circle a. The black dot denotes the centroid of the epidural hematoma, through which another diameter of the base circle passes perpendicular to the plane. Dashed lines represent a cross-section of the axial planes used to generate CT images.

A SC or spherical dome is a portion of a sphere cut off by a plane (Figure 3). The intersecting part is the base circle of the SC. Let the sphere have radius $r$, the cap have height $h$ and the base circle radius $a$, then using the Pythagorean theorem,

$r^2=a^2+\left(r-h\right)^2\tag*{(1).}$

According to this relationship, only two of the three above-mentioned parameters are required to specify a SC. The volume of a SC is given by

$V_{SC}=\frac{\pi}{3}h^2\left(3r-h\right)\tag*{(2).}$

The two-dimensional analogous object of a SC is a circular segment. When another plane (not identical to the cut-off plane) intersects with a SC, a circular segment is created. The SC has circular symmetry around an axis connecting the center of the sphere and the apex of the cap, which also passes through the center of the SC base circle. The intersection of an SC and a plane containing the base circle center in any orientation contains the radius $a$ because of such symmetry.

On an axial CT image showing the area of an EDH, the largest diameter of the EDH is always inside the biconvex hematoma area (Figure 1). If we use a circle to model the skull in that image, the area bounded by the largest hematoma diameter and the inner surface of the skull is a circular segment. Within appropriate coronal or sagittal CT images, similar circular segments can be found in areas that contain an EDH (Figure 2). Therefore, we hypothesize that the part of an EDH that is outside the cut-off plane assumes the shape of an SC.

Previously, we observed that EDHs on axial CT images are not only biconvex but also symmetric to the largest diameter of an EDH, as shown in Figure 1. Such symmetry is also noted on appropriate coronal or sagittal images. Compared to the brain parenchyma, the human dura has a much higher elastic modulus and a very small maximum strain [10]. The difficulty in expanding the surface area of the dura limits further deformation of the affected dura between the EDH and brain beyond the “reflection” of its original position across the base plane of the SC. Pressure from an epidural bleeder dissects along the inner surface of the skull instead, tearing a larger portion of the dura from its normal position and opening previously non-existent epidural space. This allows the EDH to expand. As a result, the remaining part of the EDH that is inside the cut-off plane appears to be the mirror image of the outer part, which forms another SC regardless of its volume. Moreover, the geometric centroid of the EDH occurs at the center of the base circle.

Considering an EDH within an “average” adult skull, such as the 160-mm half-sphere model we reported previously [5-7], the size and volume of the EDH can be approximated by measuring only the largest diameter $A$, which is equal to $2a$. An axial section containing the base circle diameter of the SC always exists because of its circular symmetry. In practice, contiguous axial images obtained with 5 mm spacing are sufficient for determining a good approximation of $a$ on an image containing the largest area of an EDH. The relationship between $r$, $a$, and $h$ can be rewritten as

$h=r-\sqrt{r^2-a^2} \tag*{(3).}$

If $r$ has a fixed value, such as 80 mm in our model, $h$ is determined solely by $a$. Using (2), the volume of the SC with a given $a$ and that of an EDH can be calculated using $V_{EDH\_SC}=2V_{SC}$.

Relating our model to the ABC method

Based on our half-sphere model of the supratentorial brain, we define the $X$, $Y$, and $Z$ axes of a Cartesian coordinate system as the right-left, anterior-posterior, and cranial-caudal (superior-inferior) anatomical axes, respectively [5]. The $XY$, $YZ$, and $XZ$ planes then represent the axial, sagittal, and coronal anatomical planes, respectively. The complex skull base and tentorial anatomy were simplified to become the flat basal equatorial plane of the half sphere (Figure 3).

The “ABC,” or the ellipsoid method, has been reported by Kothari et al. as being useful for measuring the volume of an ICH [11]. It can also be applied to EDH and SDH volumetry [4]. The formula for an ellipsoid is $V_E=\frac{4}{3}\pi\left(\frac{A}{2}\right)\left(\frac{B}{2}\right)\left(\frac{C}{2}\right)$, where $A$, $B$, and $C$ are the three diameters of an ellipsoid. For $\pi\approx 3$, the volume becomes $ABC/2$. In the ABC method, an axial CT image with the largest area of a hematoma is identified first. In that image, the largest diameter $A$ and the largest diameter perpendicular to $A$, $B$, is measured. $A$ and $B$ are also commonly referred to as the length and thickness of a hematoma. Then, the number of CT images showing any amount of the hematoma is counted to measure $C$, which is the cranial-caudal extent of the hematoma. Alternatively, $C$ can be measured by subtracting the $Z$ -coordinate of the most cranial hematoma image from that of the most caudal image: $C=z_{cranial}-z_{caudal}$.

As described previously, the length of the EDH that is measured using the ellipsoid method $A$ is approximately the diameter of the base circle of the SC, which is equal to $2a$. Using the same format, we define $b=B/2$ and $c=C/2$. If the axis of symmetry of an SC representing the outer half of an EDH coincides with any anatomical axis, the height of the SC, $h$, is equal to $b$ or $c$. Then the ABC method can be applied directly: $V_{EDH\_SC}=\frac{4}{3}\pi a^2h$. Since an EDH can exist anywhere underneath the skull convexity, rotating the coordinate system is required to derive $b$ and $c$ from $a$ and $h$. We constructed a working plane defined by the $Z$ axis and the axis of the SC (Figure 3). Let $\varphi$ denote the angle between the SC axis and the basal plane, or the “latitude.” It can then be measured using the $Z$-coordinate of the EDH centroid $z_{centroid}=(z_{cranial}+z_{caudal})/2$, and that of the basal plane.

$\varphi=\sin^{-1}\frac{(z_{cranial}+z_{caudal})/2-z_{basal}}{r-h}\tag*{(4).}$

For the right-angled triangle that was used to define $\varphi$, the hypotenuse is $r-h$, not $r$.

**Figure 4:**
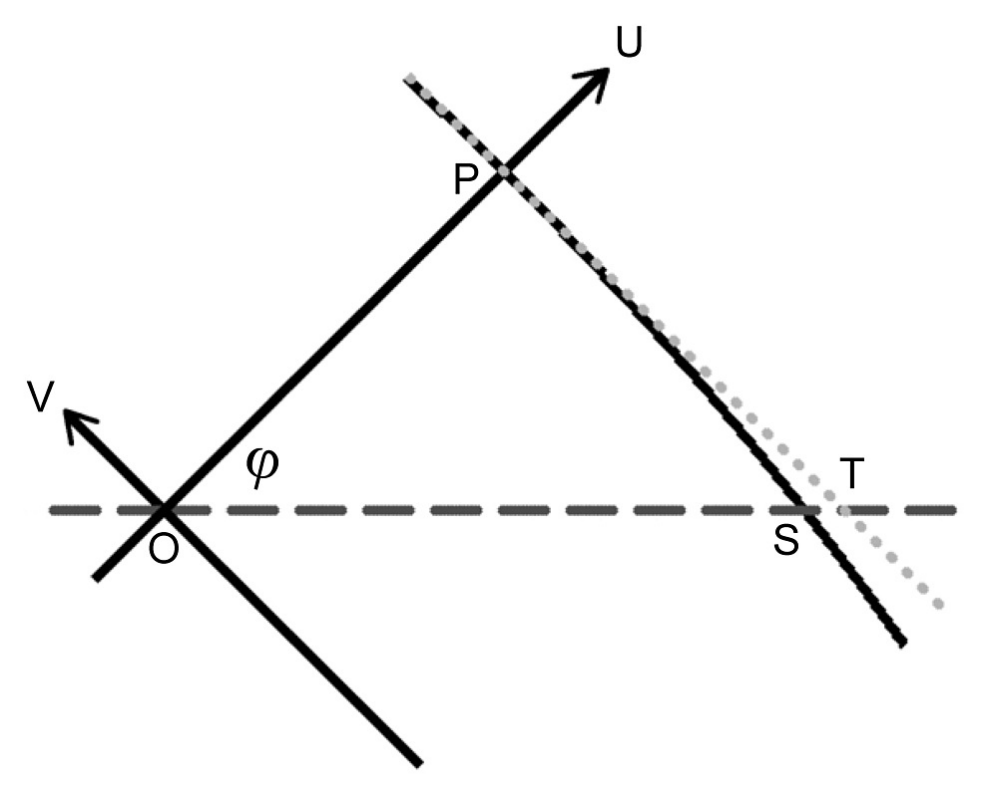
Enlarged area around the asterisk in Figure 3. Points O and P denote the center of the base circle and the apex of the spherical cap. On the axial plane (horizontal dashed line), points S and T denote the intersections with the spherical cap (arc) and the tangent line (dotted line). The U and V axes are perpendicular.

The shorter dashed line through the centroid of the EDH in Figure 3 represents the axial plane containing the largest EDH area, which is where $a$ and $b$ are measured. A simple way to estimate the hematoma thickness is using another right-angled triangle labeled with an asterisk, formed by the tangent line through the apex (dotted line in Figure 3, line segment $OT$ in Figure 4),

 $\hat{b}=h/cos\varphi\tag*{(5).}$

For smaller values of $\varphi$, this formula gives acceptable results. It can be used in most “low-lying” EDHs involving bleeders from branches of meningeal arteries originating from the skull base. Since $\cos{\varphi}\le1$, the estimation of $\hat{b}$ becomes inaccurate with large $\varphi$; i.e., an EDH near the vertex, or the most cranial point of the skull. Therefore, an exact solution for $b$ is still desirable.

To derive $b$ from $a$ and $\varphi$, we centered the two-dimensional coordinate system of Figure 3 to the EDH centroid and aligned it to the axis of the SC. The vertical and horizontal axes in Figure 3 are translated and rotated to the $U$ and $V$ axes defined by line segment $OP$ which has a length of $h$, as shown in Figure 4. The exact solution for $b$, the length of line segment $OS$, is given by the following system of equations:

\(\left\{\begin{matrix} (u-(h-r))^2+v^2=r^2 \\ v=u\tan\varphi \end{matrix}\right. \tag*{(6).}\)

The first equation represents the circle in Figure 3, whose center has the coordinate of $(h-r,0)$ in $U,V$ coordinates. By substituting the second formula into the first one, $u$ can be obtained by solving the quadratic equation

$(u+r-h)^2+(u\tan{\varphi})^2=r^2\tag*{,}$

which can be rearranged into

$\left(1+\tan^2{\varphi}\right)u^2+2\left(r-h\right)u+\left(h^2-2rh\right)=0 \tag*{.}$

In our geometric model, $u$ should be positive. Taking the positive solution, we have

$u=\frac{(h-r)+\sqrt{(h-r)^2-(\tan^2{\varphi}+1)(h^2-2rh)}}{1+\tan^2{\varphi}}\tag*{(7).}$

Furthermore, the distance between points O and S, $b$ can be expressed using $u$ , $v$ , and $\varphi$,

$b=\sqrt{u^2+v^2}=u\sqrt{1+\tan^2{\varphi}}\tag*{;}$

and subsequently using $h$, $r$ and $\varphi$,

$b=\frac{\left(h-r\right)+\sqrt{\left(h-r\right)^2-\left(1+\tan^2{\varphi}\right)\left(h^2-2rh\right)}}{\sqrt{1+\tan^2{\varphi}}}\tag*{(8).}$

From this formula, we can derive $b=h$ when $\varphi=0$ and $b=a=\sqrt{2rh-h^2}$ when $\varphi=90^{\circ}$. Although $c$ can be measured, it can also be calculated from $a$ using yet another right-angled triangle, as shown in Figure 3 when $\varphi&lt;90^{\circ}$,

$c=a\cos{\varphi}\tag*{(9).}$

When $\varphi=90^{\circ}$, $c=h$. Using (7) and (8), we can calculate $b$ and $c$ from $a$, $r$ and $\varphi$.

Application to real-life images

The skull base comprises three parts, referred to as the anterior, middle, and posterior cranial fossae [12]. On the cranial-caudal axis, the anterior fossa is closest to the vertex, followed by the middle and posterior fossae. The supratentorial space containing the cerebral hemispheres occupies the anterior and middle fossae and is separated from the posterior fossa by the cerebellar tentorium. Unlike the basal plane of our model, none of these structures is completely flat. This difference may cause errors while using the model to estimate $b$ and $c$ from $a$.

In the “native” half-sphere model, $\varphi_N=\varphi$, as defined in (4). After identifying axial CT images containing any EDH, as is usually done with the ABC method, the image containing the midbrain is identified, and its $Z$ -coordinate is taken as $z_{basal}$. There is no need to identify $z_{vertex}$ because $z_{vertex}=z_{basal}+r$ . However, the average cranial-caudal distance of the supratentorial brain in our previous craniometry study was 90 mm, which was slightly larger than that of the native model.

We aimed to correct this error by identifying the vertex; i.e., the most cranial CT image containing any brain area. Its $Z$ -coordinate was taken as $z_{vertex}$ and $r$ in (4) was replaced with $r_L=\ z_{vertex}-z_{basal}$. In the landmark-corrected model, $\varphi_L$ is defined as

$\varphi_L=\sin^{-1}{\frac{(z_{cranial}+z_{caudal})/2-z_{basal}}{z_{vertex}-z_{basal}-h}}\tag*{(10).}$

We used CT images from 15 patients with EDHs who were reported in our previous studies to verify the SC EDH model proposed in this paper [7, 8]. Our data collection process conformed to the requirements of the Human Subject Committee, Taipei Hospital, Department of Health, Taiwan, and was approved as such (TH-IRB-10-11). EDHs with atypical geometric features prohibiting successful application of the ABC method, such as multiple overlapping hematomas, interruption of the biconvexity by bony features, or fractures at the skull base, were excluded.

In these images, the diameters of the EDHs were measured and then halved to determine the radii. $Z$ -coordinates of relevant CT images (cranial, caudal, vertex, and basal) were also recorded as depicted in Figure 3. To avoid confusion, we used $a_M$ , $b_M$ and $c_M$ to denote the three manually-measured EDH radii. The height of the spherical cap was calculated by substituting $a=a_M$ into (3). After calculating $\varphi_N$ and $\varphi_L$ using (4) and (10), respectively, the estimations of $b_N$, $c_N$, $b_L$, and $b_L$ were obtained by substituting them into (8) and (9), and then comparing them to $b_N$ and $b_L$. Because both $b$ and $c$ should be $0$ when $a=0$, we employed regression through the origin to determine the relationship between the two sets of estimations and manual measurements [13].

## Results

Geometric features of our EDH model

The relationships among $A$, $h$, $V_{EDH\_ABC}$, and $V_{EDH\_SC}$ are shown in Table 1. $\varphi$ is assumed to be 0 in this table so the hematoma thickness $b$ is equal to $h$ without any need for conversion using (8). Compared to two times the SC volume, the estimated EDH volume is larger using the ABC formula, with a ratio near 1.3. According to the guideline, an EDH greater than 30 mL should be surgically evacuated regardless of the patient’s neurological condition [1]. A midline shift greater than 5 mm and a thickness greater than 15 mm on the initial CT scan were also considered as significant. Using our model, a SC height of 7.5 mm corresponds to a base diameter or a maximal EDH diameter of 67.6 mm. Using the ABC method and SC formula, a 30 mL-EDH corresponds to base diameters of 64.7 and 69.1 mm, respectively. Therefore, an EDH longer than 7 cm would occupy more than 30 mL of the intracranial volume.

**Table 1:** The relationship between the spherical cap base circle diameter (A), cap height (h), two different volume estimation methods, and their ratio in our model. Bolded values indicate treatment thresholds depicted in the guideline. This table is truncated because EDHs with larger diameters; i.e., exceeding 150 mm, have volumes larger than our half-sphere model. V_ABC: $V_{EDH\_ABC}$, 2V_SC: $V_{EDH\_SC}$

A (mm)	a (mm)	h (mm)	V_ABC (mL)	2V_SC (mL)	ratio
0.0	0.0	0.0	0.00	0.00	N/A
10.0	5.0	0.2	0.02	0.01	1.33
20.0	10.0	0.6	0.3	0.2	1.33
30.0	15.0	1.4	1.3	1.0	1.33
40.0	20.0	2.5	4.3	3.2	1.33
50.0	25.0	4.0	10.5	7.9	1.32
60.0	30.0	5.8	22.0	16.7	1.32
64.7	32.4	6.8	30.0	22.8	1.31
65.0	32.5	6.9	30.5	23.2	1.31
67.6	33.8	7.5	35.9	27.4	1.31
69.1	34.6	7.9	39.3	30.0	1.31
70.0	35.0	8.1	41.4	31.6	1.31
80.0	40.0	10.7	71.8	55.2	1.30
90.0	45.0	13.9	117.5	90.9	1.29
100.0	50.0	17.6	183.8	143.5	1.28
110.0	55.0	21.9	277.6	219.2	1.27
120.0	60.0	27.1	408.4	327.1	1.25
130.0	65.0	33.4	590.4	481.7	1.23
140.0	70.0	41.3	847.1	708.9	1.19

**Figure 5:**
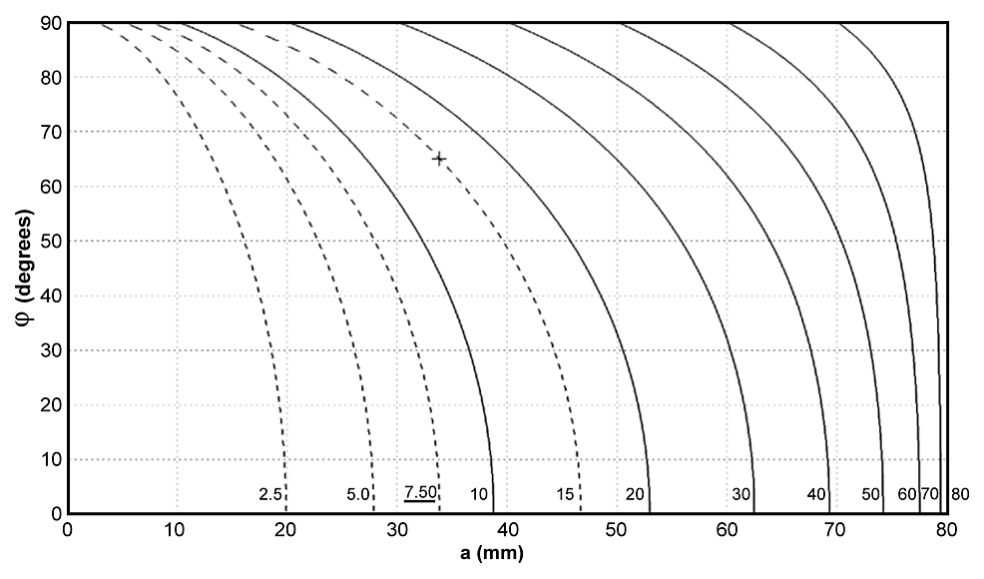
The values of b, axial hematoma thicknesses used in the ABC method, plotted against a and φ; units in mm. Irrespective of φ, points with the same a have the same spherical cap height and EDH volume. Although a 7.5 mm-hematoma thickness (underlined) is considered significant, using b instead of the true cap height can be misleading when φ is large.

For an EDH with its SC axis outside the basal plane, the thickness on the axial image with the largest hematoma area, $b$, depends on $\varphi$ and $a$, as formulated in (8). The values of $b$ plotted against $\varphi$ and $a$ are shown in Figure 5. With $\varphi=0$, $b=h$ and the axial image with the largest area of the EDH is the basal plane of the half-sphere model, as described previously. When $\varphi =90^{\circ}$, the EDH appears as a circle on the axial image with the largest area and $b=a$. The difference between $b$ and $h$ is small when an EDH is close to the basal plane, which is common for most hematomas. However, as the EDH exists closer to the vertex of the skull, $b$ overestimates $h$ significantly. For example, a 15 mm-thick EDH ( $a=33.8 mm$, $h=7.5 mm$ ) with the skull vertex lying on the circumference of its SC basal circle, $\varphi$ is approximately 65 degrees with a $b$ of 15 mm (30 mm “thickness” on the axial image, labeled on Figure 5 with a cross) instead of 7.5 (underlined value near the horizontal axis). In other words, using the EDH thickness on an axial image $b$ as the sole criterion for the guideline without calculating the volume leads to overtreatment of hematomas near the vertex.

Accuracy of predictions of BC from A

All 15 patients had one EDH on their CT images. Among them, one case was excluded because the EDH had a non-biconvex shape, partitioned by the lesser sphenoid wing protruding from the skull base. We measured the geometric features of the remaining 14 EDHs. All of them underwent surgical removal with the hematoma type confirmed. The maximal diameter $a$ of the EDHs ranged from 32.3 mm to 50.0 mm, with an average of 41.3 ± 5.8 mm. The hematoma thickness $b_M$ ranged from 8.0 mm to 25.5 mm, with an average of 15.0 ± 4.8 mm. The cranial-caudal extent $c_M$ ranged from 22.5 mm to 57.8 mm, with an average of 35.0 ± 11.1 mm. The estimated hematoma volume $V_{EDH\_ABC}$ ranged from 23.3 mL to 138.3 mL, with an average of 90.4 ± 32.6 mL.

The distance between the basal plane and that containing the EDH center, $(z_{vertex}+z_{vertex})/2-z_{basal}$, ranged from 15.1 to 70.8 mm, with an average of 43.7 ± 14.8 mm and a median of 46.1 mm. Using the native model, the angle between the spherical cap axis and the basal plane $\varphi_N$ ranged from 5.1 degrees to 58.9 degrees, with an average of 31.2 ± 14.6 degrees. The distance between the vertex and the basal plane, $z_{vertex}-z_{basal}$ , ranged from 82.5 mm to 97.8 mm, with an average of 89.6 ± 5.1 mm. The landmark-corrected angle $\varphi_L$ ranged from 11.3 degrees to 62.1 degrees, with an average of 35.2 ± 13.3 degrees. In every patient $\varphi_L$ was larger than $\varphi_N$.

**Figure 6:**
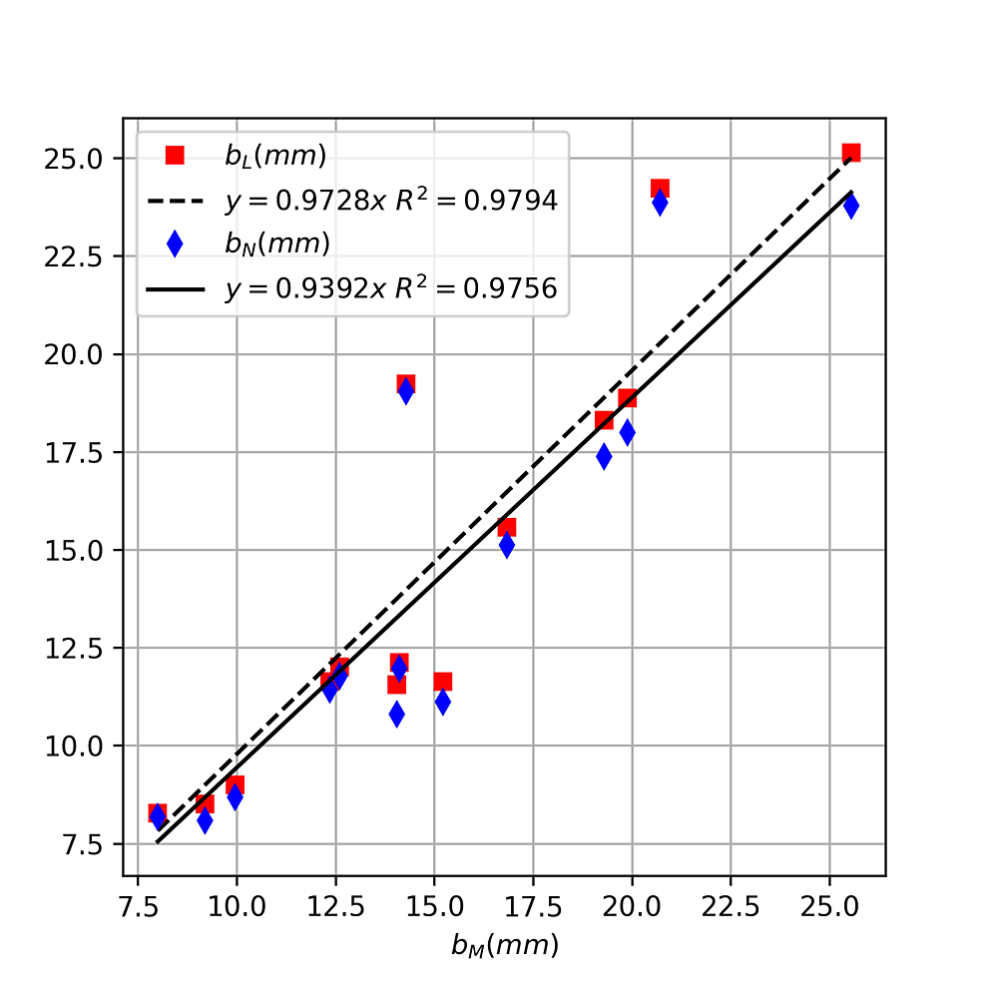
Comparison of the EDH thicknesses measured manually ( $b_M$ ) and those estimated from hematoma lengths using models that employed native ( $b_N$ ) and landmark-corrected angles ( $b_L$ ).

The values for $b_N$, the EDH thickness estimation based on $a$ and $\varphi_N$, ranged from 8.1 mm to 23.9 mm, with an average of 14.0 ± 5.3 mm; while $b_L$ , the estimation based on $\varphi_L$ , ranged from 8.5 mm to 25.1 mm, with an average of 14.5 ± 5.5 mm. The absolute difference for $b_M$ and $b_N$ ranged from 0.5 mm to 4.8 mm, with an average of 2.2 ± 1.3 mm; while that between $b_M$ and $b_L$ ranged from 0.4 mm to 5.0 mm, with an average of 1.8 ± 1.4 mm. Using t-tests for paired data sets, we could not detect statistically significant differences between $b_M$ and $b_N$ (p = 0.16) or between $b_M$ and $b_L$ (p = 0.43). Using linear regression through the origin, we obtained a slope of 0.9392 ( $R^2$ =0.9756) for $b_N$ and a slope of 0.9728 ( $R^2$ =0.9794) for $b_L$ (Figure 6). The largest discrepancy occurred in a patient with a heterogeneous hematoma, indicating active bleeding with ongoing hematoma expansion.

**Figure 7:**
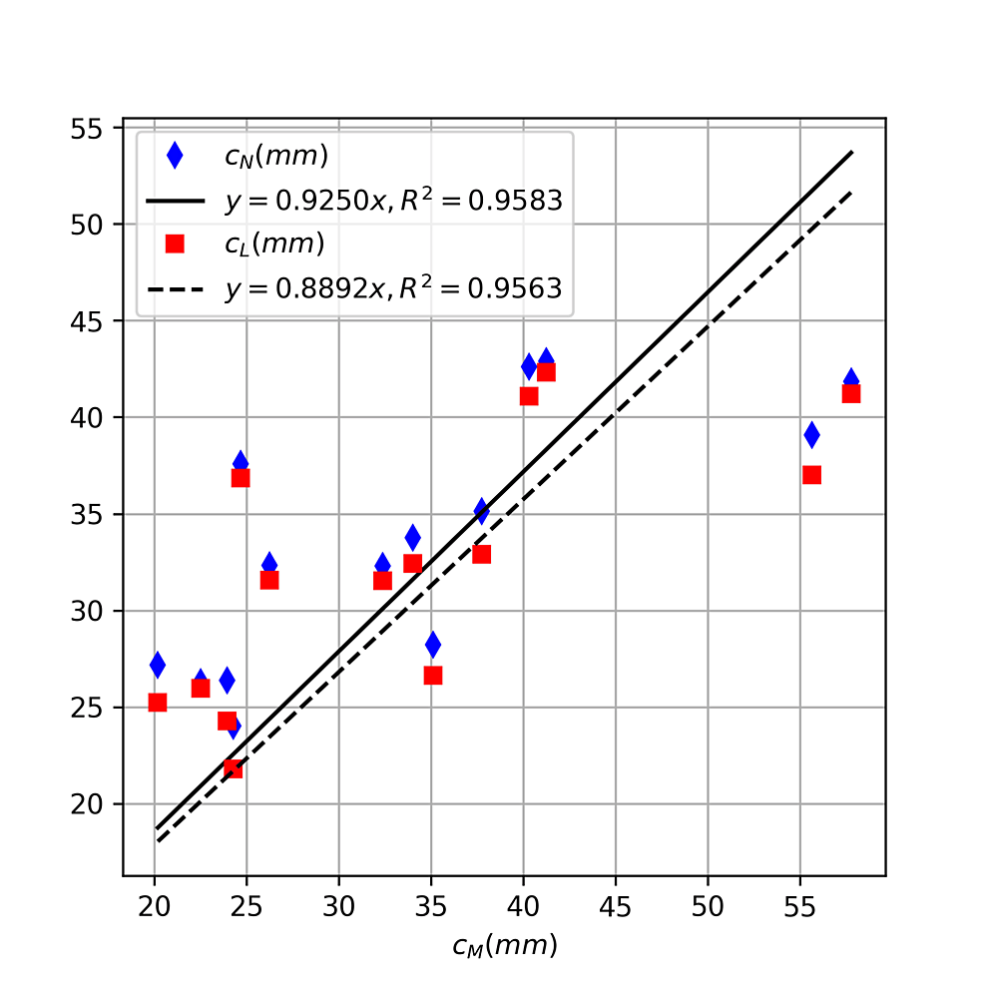
Comparison of cranial-caudal EDH extents measured manually ( $c_M$ ) and those estimated from hematoma lengths using models that employed native( $c_N$ ) and landmark-corrected angles ( $c_L$ )

The values for $c_N$, the estimation of cranial-caudal EDH extent based on $a$ and $\varphi_N$, ranged from 24.0 mm to 42.9 mm, with an average of 34.1 ± 6.4 mm, while $c_L$, the estimation based on $\varphi_L$, ranged from 21.8 mm to 42.3 mm, with an average of 32.7 ± 6.6 mm. The absolute difference for $c_M$ and $c_N$ ranged from 0.03 mm to 16.5 mm, with an average of 5.1 ± 5.8 mm; while that between $c_M$ and $c_L$ ranged from 0.4 mm to 18.6 mm, with an average of 5.5 ± 6.1 mm. Using t-tests for paired data sets, we could not detect statistically significant differences between $c_M$ and $c_N$ (p = 0.65) or between $c_M$ and $c_L$ (p = 0.29). Using linear regression through the origin, we obtained a slope of 0.9250 ( $R^2$ =0.9583) for $c_N$ and a slope of 0.8892 ( $R^2$ =0.9563) for $c_L$ (Figure 7).

## Discussion

The “ABC/2” method (or “ABC method” for short) was widely applied in estimating the volume of post-traumatic masses, including EDHs, SDHs, and ICHs [4]. However, this method was based on the ellipsoid model, while EDHs are usually in biconvex shapes. It is therefore that the ABC/2 method may overestimate EDH volumes. Based on CT scans of 35 EDH patients, Hu et al. compared the planimetric method with the ABC/2 method and claimed that the ABC/2 method usually overestimates the EDH volumes [14]. The difference may be significant, especially in the subjects with nonellipsoid-like EDHs, where a mean difference of 36.3% was reported. 

Yan et al. also compared the planimetric method with four variants of the ABC method in traumatic EDH volume estimation on 53 subjects [15]. Their results showed that, without adjustment, variants of the ABC method overestimated EDH volumes, with overall deviations ranging from 32.73% to 40.29%. 

There was another method to estimate the hematoma volume using the area value of the largest axial hematoma slice, and this method was shown accurate for EDH patients [16]. However, measurement of the area value itself is already difficult without the assistance of computer algorithms, so this method was not popular in clinical practice. 

In this paper, we proposed a geometric model of EDH using two SCs. To the best of our knowledge, this is the first such geometric model. With our model, the volume of a given EDH is readily available once its largest diameter is measured. However, the volume derived with the SC formula was found to be different from the ABC method, with the latter always being larger. By substituting $a^2$ with $2rh-h^2$ into the standard ellipsoid formula $V_{EDH\_ABC}=\frac{4}{3}\pi\left(a^2h\right)$, it becomes

$V_{EDH\_ABC}=\frac{4}{3}\pi h^2\left(2r-h\right)=\frac{2}{3}\pi h^2\left(4r-2h\right) \tag*{.}$

Compared to (2), we have $\frac{V_{EDH\_ABC}}{V_{EDH\_SC}}=\frac{4r-2h}{3r-h}$. This ratio is close to $4/3$ when $h$ is very small. With increasing hematoma sizes, the ratio gradually decreases but remains larger than 1.2 for most EDHs. $V_{EDH\_ABC}=V_{EDH\_SC}$ only when $h=r$ , but this hematoma size cannot exist clinically. We also noted that the computation based on our model is compatible with existing literature, where the ABC method may overestimate EDH volumes by more than 30% [15]. Although a 30% overestimation seems significant, the difference is less pronounced in practice, as shown in Table 1. The difference between the largest hematoma diameter with a $V_{EDH\_ABC}$ of 30 mL and that with a $V_{EDH\_SC}$ of 30 mL is merely 4.4 mm, which is relatively small in a hematoma longer than 60 mm [1]. Moreover, an EDH can evolve rapidly, showing considerable hematoma expansion within minutes. Many patients are clinically normal initially before devastating EDHs occur [17]. Allowing some overestimation in volume may alert physicians. In addition, large hematomas are less likely to be perfectly ellipsoid or assume the shape of SCs, decreasing the accuracy of both formulas. More volumetric studies are needed to see which estimation formula works better.

In addition to the volume, the thickness of an EDH has been proposed as another parameter to determine whether to remove it. However, we have demonstrated that hematoma thickness on an axial image $b$ can be several times that of $h$, as shown in Figure 5. Since $h\le b\le a$, $b/h$ ranges from 1 to $a/h$, which have values of 15.9, 7.9, 5.1, 3.7 and 2.8 when $a$ equals 10, 20, 30, 40, and 50 mm, respectively. Although most EDHs have bleeders at the main branches of a middle meningeal artery close to the base of the skull [1], using $b$ to replace the “true EDH thickness” $h$ causes a much larger error for those near the vertex. On the other hand, an axial image containing the base circle diameter $A$ always exists. We therefore recommend the EDH length as a better one-dimensional feature than its thickness, and its length should be employed in future guidelines. While SDHs are also extra-parenchymal intracranial hematomas, they differ from EDHs in their propensity to extend across the entire surface of the brain. Consequently, the thickness of SDHs emerges as a more indicative feature of their severity, along with the midline shift [5]. Because the volume threshold for surgery of EDHs is considerably smaller than that of SDHs, whether the midline shift has an additional contribution in addition to the EDH volume remains unclear.

Our half-sphere model represents the cerebral hemispheres above a flattened basal plane. Viewed from the vertex, the anterior, middle, and posterior cranial fossae floors are progressively deeper. Part of the temporal lobes near the middle fossa floor are caudal to the basal plane containing the midbrain. In our previous study, about 13% of the supratentorial brain volume was located caudal to the midbrain [5]. EDHs overlying this portion of the brain can have $\varphi&lt;0$. Our formulas (8) and (9) can still be used to obtain positive $b$ and $c$ values when $-90^{\circ}&lt;\varphi&lt;90^{\circ}$. In this study, we also measured the distance between the basal plane and that containing the base of the temporal lobe, which ranged from 97.5 mm to 125.1 mm, with an average of 111.6 ± 8.3 mm. However, using the middle fossa floor instead of the midbrain as the landmark of the basal plane caused excessive distortion of the model and less accurate estimations of $b$ and $c$.

Our estimation formulas for the thickness of an EDH on axial images ($b$ ) and cranial-caudal EDH extent ($c$ ) were reasonably accurate. They were not affected by the hematoma size or location. Both EDH patients with errors of $b_N$ and $b_L$ larger than 20% had radiologic signs of active bleeding. Therefore, the hematoma shape may have been changing instead of having a well-formed biconvex shape. Compared to $b$, estimation errors of $c$ were larger. Our axial images were non-volumetric with spacing between images of at least 5 mm. Such sparse sampling in the $Z$-axis compared to much better resolution within the $XY$-plane resulted in discrete values of $c$ instead of nearly continuous values of $b$. In addition, a partial volume effect inevitably affects identifying landmarks and defining their $Z$-coordinates. It is possible that $c_N$ and $c_L$ were correct estimates, while measurement of $c_M$ was limited by discretization.

Although our SC model of EDH is reasonably accurate, it has limitations. It is based on the craniometry of the supratentorial region, so its accuracy in other regions of the brain and pediatric patients is uncertain. EDHs also appear in the posterior fossa, or the infratentorial compartment of the intracranial compartment [1]. The volume of this compartment is considerably smaller than that of the supratentorial component [18], and children have smaller heads and thus smaller intracranial volumes. Formulae to compute $\varphi$, $b$, and $c$ are relatively complex and cannot be manually estimated. Errors can also occur during manual recognition of key landmarks. Although in our model, the diameters of an EDH measured on axial and coronal cuts should be the same, they can be different during clinical practice. If large discrepancies among measurements in different directions are encountered, confusion would arise. However, we suggest that the largest value should be taken into account for clinical decisions.

## Conclusions

We constructed a SC model for EDHs that describes the relationships of the three volumetric diameters. These formulas have been verified with patient images. Our model allows reducing the number of parameters to be measured in the ABC method from three to one, the hematoma length. We recommend to use the length of 7 cm as a threshold for surgical evacuation of EDHs. Such simplification accelerates clinical decision-making for critically injured patients. Further validation across diverse patient populations will enhance the generalizability and utility of this geometric modeling approach in clinical settings.
